# Long noncoding RNA CASC2 suppresses esophageal squamous cell carcinoma progression by increasing SOCS1 expression

**DOI:** 10.1186/s13578-019-0353-4

**Published:** 2019-11-09

**Authors:** Ke Sun, Guangping Zhang

**Affiliations:** Department of Oncology, The First Affiliated Hospital, and College of Clinical Medicine of Henan University of Science and Technology, No. 24 of Jinghua Road, Jianxi District, Luoyang, 471003 Henan China

**Keywords:** SOCS1, Protein stability, ceRNA, Drug sensitivity

## Abstract

**Objective:**

Esophageal squamous cell carcinoma (ESCC) is one of the leading causes of cancer-related deaths worldwide. Emerging evidence suggests the involvement of long noncoding RNAs (lncRNAs) in tumorigenesis. LncRNA Cancer Susceptibility Candidate 2 (CASC2) has been demonstrated to act as a tumor suppressor contributing to the development and progression of several cancers. However, the functional significance and underlying mechanism of CASC2 in ESCC progression has not been well elucidated.

**Methods:**

The expression levels of CASC2 in ESCC tissues were detected by qRT-PCR. CASC2 overexpression and knockdown models were established and used to investigate the functional role of CASC2 in ESCC cells. RIP, RNA pull-down and dual-luciferase assay was used to detect the association between CASC2 and miR-155. The interaction between CASC2 and Suppressor Of Cytokine Signaling 1 (SOCS1) was assessed by RIP and RNA pull-down assays.

**Results:**

In the present study, we found that CASC2 was significantly downregulated in ESCC tissues and positively correlated with overall survival time of patients with ESCC. Functional assays demonstrated that CASC2 suppressed proliferation, migration and invasion, as well as enhanced drug sensitivity in ESCC cells. Mechanistically, CASC2 inhibited ESCC progression by upregulating the expression of SOCS1 via two different ways. CASC2 acted as competing endogenous RNA (ceRNA) for miR-155 to post-transcriptionally increase SOCS1 expression. On the other hand, CASC2 was capable of interacting with SOCS1 protein and suppressing its degradation.

**Conclusion:**

Conclusively, these results demonstrated that CASC2 could exert as a tumor suppressive lncRNA in ESCC progression via regulating SOCS1.

## Introduction

Esophageal squamous cell carcinoma (ESCC) arises from esophageal epithelial cells, which is one of the main subtypes of esophageal cancer and one of the leading causes of cancer-related deaths worldwide [1]. To date, advancements in treatment of ESCC, such as surgery, chemotherapy and radiotherapy, have been achieved and improves the prognosis of patients with ESCC [2]. However, once distant metastasis or recurrence occurs, surgical resection will not be applicable, and the survival of ESCC patients will be extremely poor [3]. Hence, it is urgently needed to a better understanding of the molecular mechanisms underlying ESCC progression.

Long non-coding RNAs (lncRNAs) are defined as a class of transcripts larger than 200 nucleotides but cannot encode proteins [4]. Increasing evidence indicates that lncRNAs play important roles in human cancers, including ESCC. lncRNAs exert their function through diverse mechanisms. For example, lncRNA Prostate Androgen-Regulated Transcript 1 (PART1) promotes gefitinib resistance and can be incorporated into exosomes and transmitted to sensitive cells by competitively associating with miR-129 and upregulating Bcl-2 expression in ESCC cells [5]. LINC00312 directly interacts with the transcription factor Y-Box Binding Protein 1 (YBX1) to facilitate migration, invasion and vasculogenic mimicry of lung cancer cells [6]. Linc02023 expression is significantly decreased in colorectal cancer. Lnc02023 acts as a tumor suppressor which inhibits cell proliferation in colorectal cancer by apoptosis promotion and cell cycle rearrangement. Mechanistically, Linc02023 specifically binds to PTEN and blocks its interaction with WWP2, thus suppressing the degradation of PTEN and its downstream expression [7].

Cancer susceptibility candidate 2 (CASC2) is a lncRNA located on chromosome 10q26, which is frequently found to be downregulated in several cancers, such as pancreatic carcinoma, gastric cancer and papillary thyroid cancer [8–10]. Recent studies have shown that the restoration of CASC2 expression significantly suppresses growth and metastasis in breast cancer cells via regulation of the miR-96-5p/SYVN1 pathway and inactivation of the TGF-β signaling pathway [11, 12]. Moreover, CASC2 serves as a “sponge” of miR-24 and miR-221 to regulate the TNF Related Apoptosis Inducing Ligand (TRAIL) resistance of hepatocellular carcinoma cells [13]. However, the specific role and molecular mechanisms of CASC2 in ESCC remain unclear.

In the present study, we aimed to investigate the functional role of CASC2 in ESCC cells and its underlying mechanism. Our findings showed that CASC2 was significantly downregulated in ESCC tissues. Functionally, CASC2 suppressed proliferation, migration, invasion and drug resistance in ESCC cells. Mechanistically, CASC2 inhibited ESCC progression through post-transcriptionally and post-translationally upregulating SOCS1 expression. Our study discovered a novel CASC2-SOCS1 regulatory pathway in ESCC progression, which provided a promising therapeutic strategy for ESCC patients.

## Materials and methods

### Tissue samples

78 pairs of primary ESCC and adjacent normal tissues were obtained from 78 patientswho underwent surgery at The First Affiliated Hospital of Henan University of Science and Technology between 2012 and 2016 and diagnosed with ESCC based on histopathologic evaluation. The present study was approved by the Ethics Committee of The First Affiliated Hospital of Henan University of Science and Technology and signed informed consent from all patients was obtained. None of the recruited patients received other treatment before or after surgery.

### Cell culture and transfection

ESCC cell lines, KYSE30, KYSE70, KYSE150, KYSE180, KYSE410 and EC109, were cultured in Dulbecco’s modified Eagle’s medium (DMEM) containing 10% fetal calf serum (Hyclone) at 37 °C in a 5% CO_2_ humidified incubator. Cell transfection was performed using Lipofectamine 2000 (Invitrogen) according to the manufacturer’s instructions. The double-stranded miR-155 mimic, inhibitor and their respective negative control RNAs (Ribobio, China) were transfected into cells at a final concentration of 50 nM. The cells were harvested 48 h after transfection.

### Overexpression and RNA interference

The lentiviral particles expressing full-length CASC2 or CASC2 shRNA or SOCS1 shRNA and corresponding negative control (NC) were purchased from GenePharma (Shanghai, China). Cells were infected with above lentiviral particles by using Polybrene (5 μg/ml). After transfection, stable cells were selected with puromycin for 1 week. The infection efficiency was validated by Real-time PCR.

### Real-time PCR

Total RNA was extracted using Trizol reagent (Invitrogen, CA) according to the manufacturer’s instructions. The cDNA was synthesized using the cDNA Reverse Transcription Kit (ABI 7900; Life Technologies). Real-time PCR (RT-PCR) was performed using SYBR Green PCR kit (Takara, Dalian, China). The primers were listed as follow: CASC2-forward: GCTCTGTGCCACATTCCTAA, CASC2-reverse: GCTTTCCTCTCTTTCTCCTACC; SOCS1-forward: CCTCCTCTTCCTCCTCCTC, SOCS1-reverse: AACGGAATGTGCGGAAGT; GAPDH-forward: GTCAACGGATTTGGTCGTATTG, GAPDH-reverse: CCGTTCTCAGCCATGTAGTT. GAPDH was used as endogenous control. Relative expression level was normalized to control group and calculated by 2^−∆∆CT^ method.

### RNA sequencing

The RNA isolated from control and CASC2-overexpressed KYSE30 cells was quantified and its quality was assessed by Agilent 2100 Bioanalyzer (Agilent). mRNA was then enriched using oligo(dT) beads, fragmented and then reverse transcribed into cDNA using random primers. Second-strand cDNA was synthesized using DNA polymerase I, RNase H, dNTPs, and buffer. Next, poly(A) tails were added to the cDNA fragments, and the fragments were ligated with Illumina sequencing adapters. Ligation products were selected according to size by performing agarose gel electrophoresis, amplified by PCR, and sequenced using Illumina HiSeq™2500. Differential mRNAs were screened based on a fold change of ≥ 2 and a p value of < 0.05.

### Isolation of cytoplasmic and nuclear

Cytoplasmic and nuclear RNA were isolated and purified using the Cytoplasmic & Nuclear RNA Purification Kit (Norgen, Belmont, CA) according to the manufacturer’s instructions.

### Cell proliferation detection

Cell proliferation was determined by CCK-8 and colony formation assay. For CCK-8 assay, 3 × 10^3^ cells per well were seeded in a 96-well plate. At indicated time point, the CCK-8 solution was added to each well and incubated for 1 h. The relative number of cells was assessed by measuring the optical density (OD) values at 450 nm and normalized to time 0. CCK-8 allows sensitive colorimetric assays for the determination of cell viability in cell proliferation and cytotoxicity assays. The highly water-soluble tetrazolium salt, WST-8, is reduced by dehydrogenase activities in cells to give a yellow-color formazan dye, which is soluble in the tissue culture media. The amount of the formazan dye, generated by the activities of dehydrogenases in cells, is directly proportional to the number of living cells. For colony formation assay, cells were seeded at a density of 2000 cells per well in six-well plates and incubated for 12 days. Cells were subsequently fixed with 10% methanol and stained with 0.1% crystal violet.

### In vitro drug sensitivity assay

Cells were seeded at a density of 2000 cells per well in 96-well plates, freshly prepared medium containing several final concentrations of cisplatin or capecitabine was added to the wells, with three replicate wells for each concentration. After incubation for an additional 48 h, cell viability was measured using CCK-8 (Dojindo, Japan), according to the manufacturer’s instructions.

### Cell apoptosis analysis

Cells were treated with cisplatin or capecitabine for 24 h, and then were diluted to 1 × 10^6^/ml in 500 μl with binding buffer. Cells were then stained with Annexin V (5 μl) and PI (5 μl) for 15 min. Apoptosis was analyzed by FITC Annexin V Apoptosis Detection Kit (Becton–Dickinson, NJ, USA).

### Transwell assay

To detect the cell migration and invasion, transwell assay was utilized. Indicated cells were cultured with 200 μl serum-free DMEM in the upper chamber (Millipore) without (for cell migration detection) or with Matrigel (for cell invasion detection). DMEM supplemented with 10% fetal bovine serum was added in the lower chamber and incubated at 37 °C for 48 h. Then, cells on the lower membrane surface were fixed with 10% methanol and stained with 0.1% crystal violet, and then counted under a microscope in five random fields.

### Western blot

Cells were lysed in RIPA buffer with protease inhibitors and phosphatase inhibitors. Protein was loaded onto an SDS-PAGE minigel and transferred onto a PVDF membrane. The blots were probed with anti-SOCS1 (Abcam, MA, USA) or anti-GAPDH (Abcam, MA, USA) antibody followed by the HRP-conjugated secondary antibody. Signals were visualized using ECL Substrates (Millipore). GAPDH served as the loading control.

### RNA immunoprecipitation (RIP)

RIP assay was performed using the Magna RIP RNA-Binding Protein Immunoprecipitation Kit (Millipore, Bedford, MA) according to the manufacturer’s instructions. To detect the interaction between CASC2 and SOCS1, cells were lysed in lysis buffer containing a protease inhibitor cocktail and RNase inhibitor. Cell lysates were incubated with magnetic beads and anti-SOCS1 antibodies or negative control anti-rabbit IgG at 4 °C overnight. RNA was purified from RNA–protein complexes bound to the beads and was then analyzed by Real-time PCR. MS2-binding protein (MS2 bp) specifically binds RNA containing MS2-binding sequences (MS2bs). To detect the interaction between CASC2 and miR-155, KYSE30 and KYSE150 cells were co-transfected with pcDNA3.1-MS2, pcDNA3.1-MS2-CASC2, or pcDNA3.1-MS2-CASC2-MUT and pMS2-GFP (Addgene). After 48 h, cells were used to perform RIP experiments using GFP antibody (Millipore) as described above. For anti-AGO2 RIP, KYSE30 and KYSE150 cells were transfected with miR-155 or microRNA negative control (miR-NC). After 48 h, cells were used to perform RIP experiments using AGO2 antibody (Millipore) as described above.

### RNA pull-down assay

RNA pull-down assay was performed as described previously [14]. The pSPT19 plasmid containing sense or antisense CASC2 were constructed and purchased from GenePharma Company. Briefly, RNAs were biotin-labeled and in vitro transcribed. After purification, biotinylated RNAs were mixed and incubated with ESCC cell lysates. Streptavidin agarose beads (Life Technologies) were added to each binding reaction and incubated for 1 h. The beads were then boiled in sodium dodecyl sulfate (SDS) buffer. The eluted proteins were detected by western blot.

### Luciferase reporter assays

Cells were co-transfected with luciferase reporter vector comprising the wild type or mutant CASC2 fragment (CASC2-WT or CASC2-MUT) and miR-NC or miR-155. Luciferase activities were measured 48 h post-transfection using the Dual-Luciferase Reporter Assay System (Promega, Madison WI, USA).

### Statistical analysis

All statistical analyses were performed using SPSS software (Abbott Laboratories, Chicago, IL). All of the relative values were normalized to control group. Survival curves were calculated using Kaplan–Meier and log-rank tests. The Chi square test was used to analyze the relationship between CASC2 expression and clinicopathological features of ESCC patients. Student’s *t* test or multi-way classification ANOVA tests were performed for results from Real-time PCR, colony formation assay, CCK-8 assay and tumor growth curve detection. Correlations between CASC2 and SOCS1 were analyzed by Pearson correlation. The results were considered statistically significant at p < 0.05.

## Results

### LncRNA CASC2 is downregulated in ESCC tissues

**Fig. 1:**
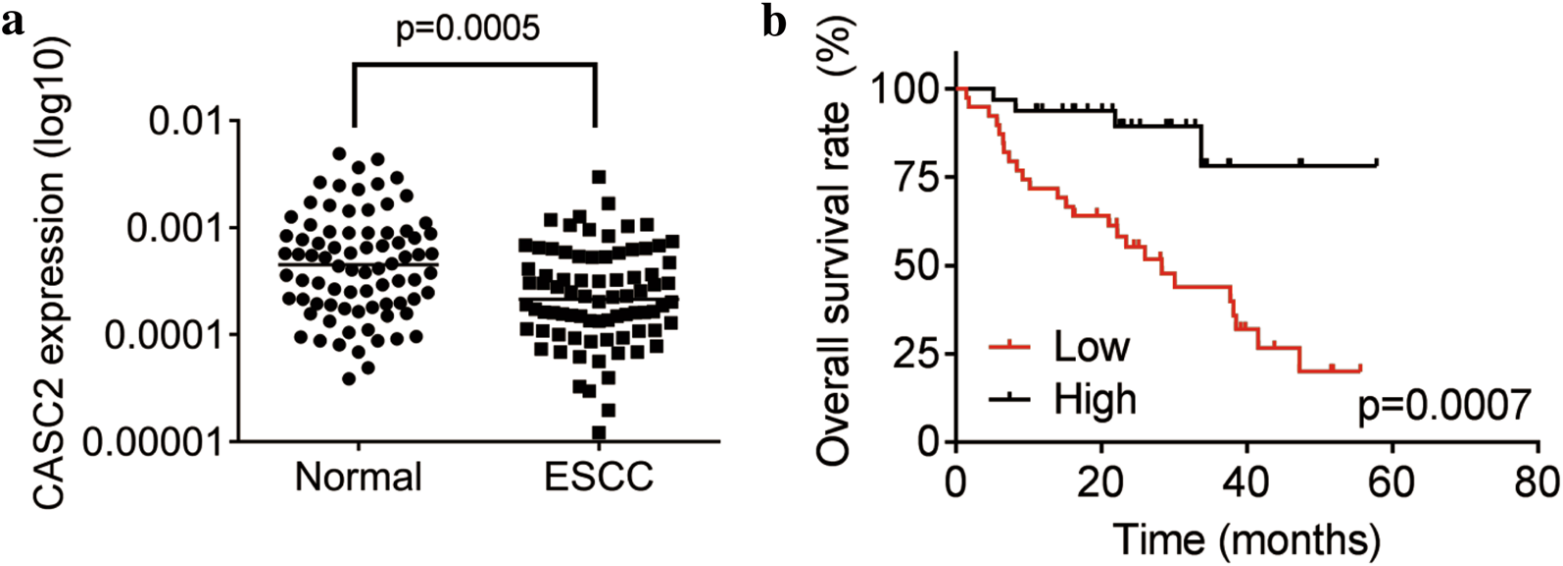
Downregulation of CASC2 expression in ESCC tissues predicts poor prognosis. **a** The expression levels of lncRNA CASC2 in 78 pairs of ESCC tissues and adjacent normal tissues were detected by using real-time PCR. **b** Kaplan–Meier plots of ESCC patients with high and low levels of CASC2. The median of CASC2 expression in ESCC tissues was used as cutoff

We first assessed the expression pattern of CASC2 in ESCC tissues. We measured CASC2 expression in 78 pairs of ESCC tissues and adjacent normal tissues using real-time PCR. As shown in Fig. 1a, CASC2 expression was significantly decreased in 88.7% (68/78) of ESCC tissues compared to their corresponding adjacent normal tissues. To determine whether CASC2 expression levels were related to the ESCC progression, we analyzed the relationship between CASC2 and clinicopathological features of ESCC patients. We found that CASC2 levels were significantly associated with tumor differentiation, lymph node metastasis, and TNM stage, but not correlated with age, gender, tumor size, smoking status and tumor location (Table 1). Furthermore, Kaplan–Meier analysis was utilized to analyze the correlation between survival time of ESCC patients and CASC2 expression. Obviously, the result indicated that the low expression group had the shorter survival time (Fig. 1b), strongly suggesting that downregulation of CASC2 expression might contribute to ESCC progression.

**Table 1:** Correlation between CASC2 expression and clinical features of ESCC patients

Characteristics	CASC2 expression		p value
	Low	High	
Age (years)			0.337
≤ 60	28	24	
> 60	11	15	
Gender			0.651
Male	19	21	
Female	20	18	
Smoking status			0.482
No	13	16	
Yes	26	23	
Tumor Size			0.651
≤ 3 cm	18	20	
> 3 cm	21	19	
Tumor location			0.821
Upper	19	20	
Middle + lower	20	19	
TNM stage			0.003
I + II	9	22	
III + IV	30	17	
Lymph node metastasis			0.013
No	13	25	
Yes	26	16	
Differentiation			0.022
Low	12	22	
Moderate and high	27	17	

The median of CASC2 expression in ESCC tissues was used as cutoff. Pearson x^2^ test

### CASC2 suppresses ESCC cell proliferation, migration and invasion

**Fig. 2:**
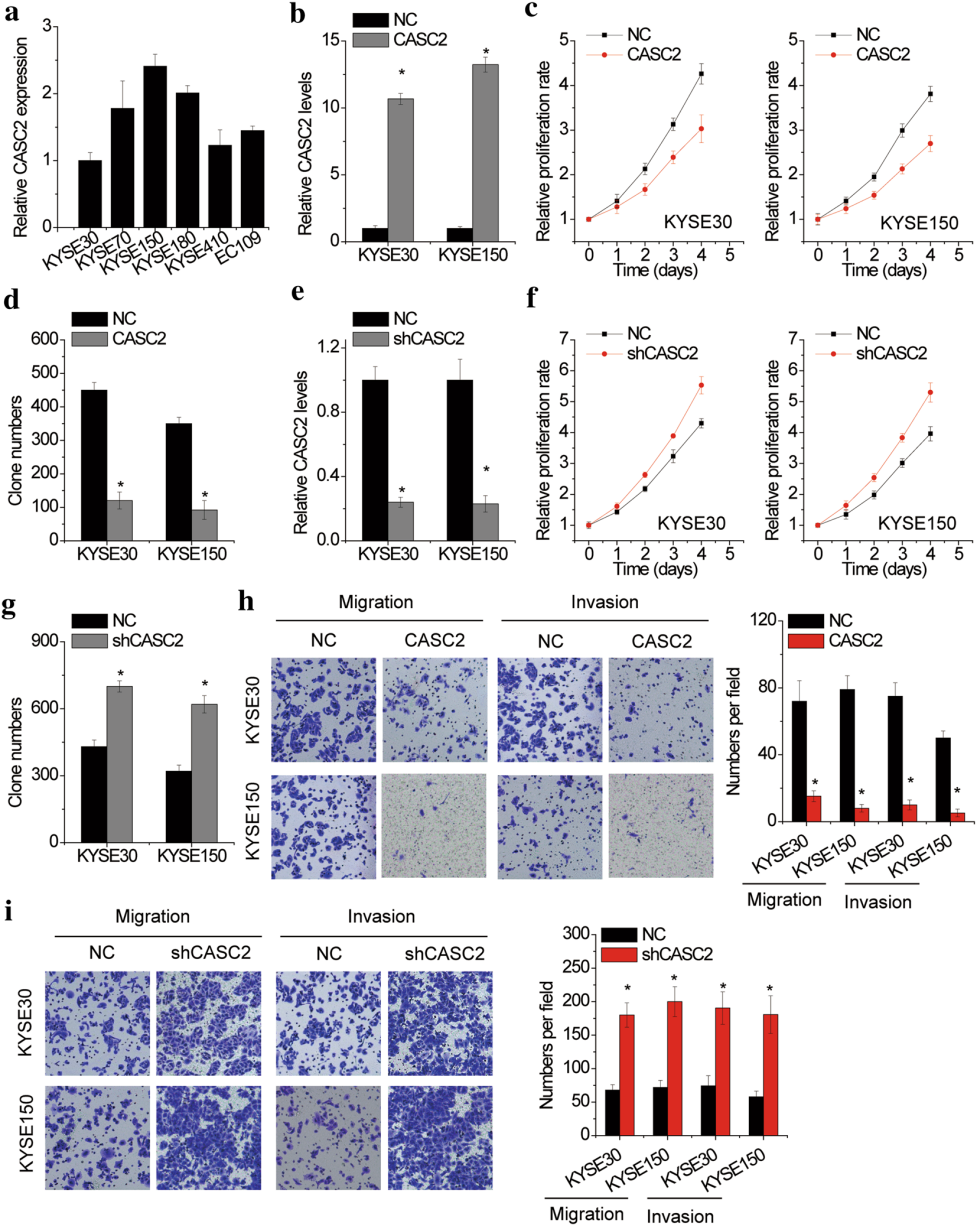
CASC2 suppresses ESCC cell proliferation, migration and invasion. **a** The expression levels of lncRNA CASC2 in different ESCC cell lines. **b** CASC2 was overexpressed in KYSE30 and KYSE150 cells. The CASC2 overexpression efficacy was confirmed by real-time PCR. **c** The effect of CASC2 overexpression on the proliferation of KYSE30 and KYSE150 cells was detected by CCK-8 assay. **d** The colony formation number of control and CASC2 overexpressing KYSE30 and KYSE150 cells. **e** CASC2 was knocked down in KYSE30 and KYSE150 cells. The CASC2 knockdown efficacy was confirmed by real-time PCR. **f** The effect of CASC2 knockdown on the proliferation of KYSE30 and KYSE150 cells was detected by CCK-8 assay. **g** The colony formation number of control and CASC2 silencing KYSE30 and KYSE150 cells. **h** Migration and invasion assays of KYSE30 and KYSE150 cells with CASC2 overexpression by Transwell assay (quantification in right histogram). **i** Migration and invasion assays of KYSE30 and KYSE150 cells with CASC2 knockdown by Transwell assay (quantification in right histogram). Error bars indicate SD. *p < 0.05

Expression of CASC2 was detected in six ESCC cell lines (KYSE30, KYSE70, KYSE150, KYSE180, KYSE410 and EC109). KYSE30 and KYSE150 cell lines with relatively low and high expression of CASC2 were selected in the functional experiments (Fig. 2a). To investigate the biological role of CASC2 in ESCC malignant phenotypes, CASC2 expression was overexpressed in KYSE30 and KYSE150 cells by transfection of lentiviral particles expressing full-length CASC2 (Fig. 2b). The results of CCK-8 and colony formation assays demonstrated that overexpression of CASC2 decreased the proliferative ability of KYSE30 and KYSE150 cells, compared to that of parallel stable cell lines containing the empty vector (Fig. 2c, d, Additional file 1: Figure S1A). Conversely, we developed stable KYSE30 and KYSE150 cells lwith CASC2 silence (Fig. 2e). It was observed that depletion of CASC2 enhanced the cellular proliferation in KYSE30 and KYSE150 cells compared to the control groups (Fig. 2f, g, Additional file 1: Figure S1B).

Given that CASC2 expression was associated with metastatic potential of ESCC (Table 1), we further confirmed the metastasis inhibition by CASC2. Transwell assays were conducted to understand the functional role of CASC2 in ESCC cell migration and invasion. The results indicated that CASC2 overexpression reduced the cell migration and invasion, while knockdown of CASC2 enhanced the migration and invasion capacity in KYSE30 and KYSE150 cells (Fig. 2h, i).

### CASC2 enhances drug sensitivity of ESCC

**Fig. 3:**
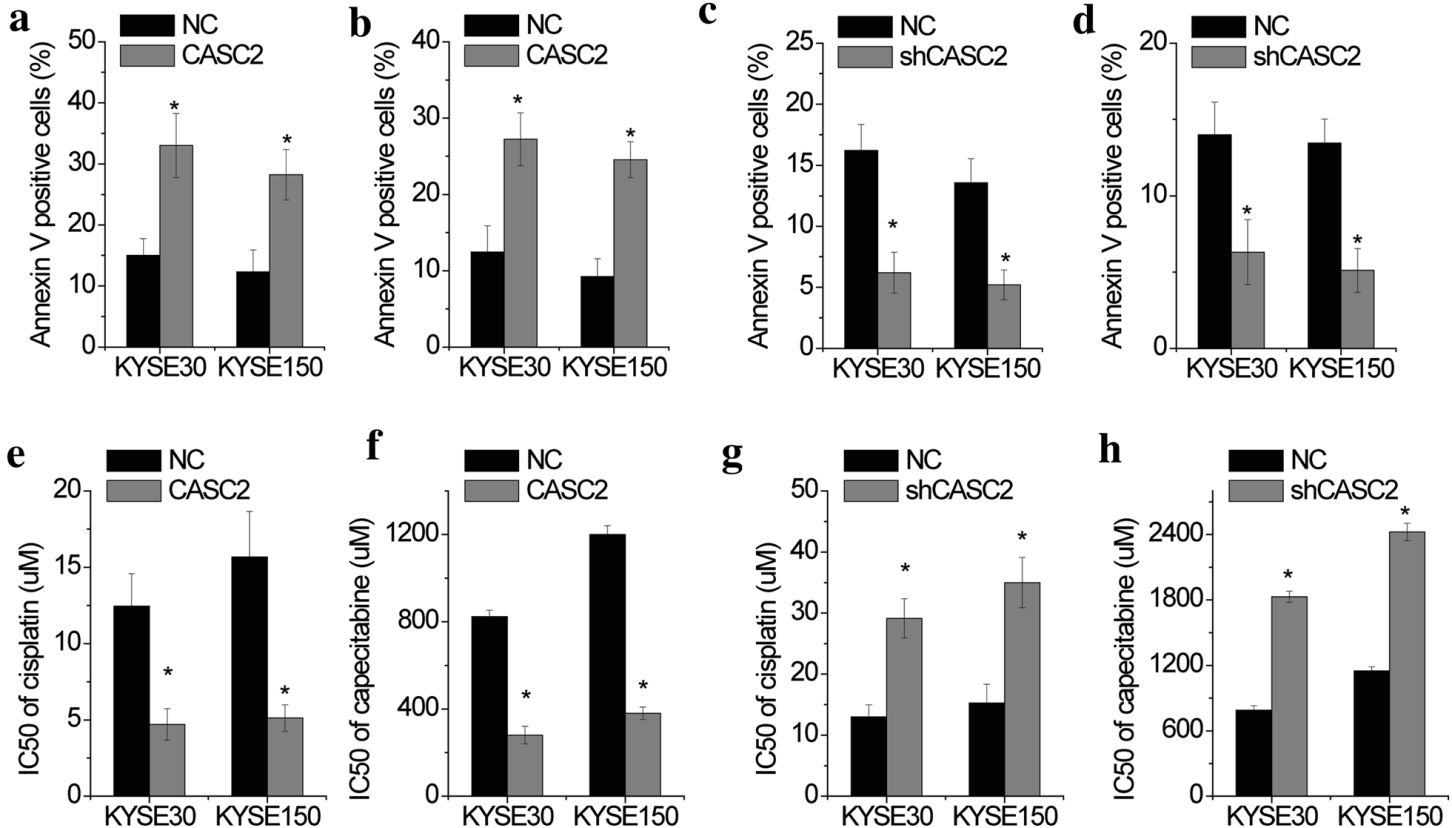
CASC2 promotes the drug sensitivity of ESCC cells. Control and CASC2 overexpressing KYSE30 and KYSE150 cells were treated with 10 μM cisplatin (**a**) or 800 μM capecitabine (**b**) for 24 h. The cell apoptosis was detected by flow cytometry. Control and CASC2 knockdown KYSE30 and KYSE150 cells were treated with 10 μM cisplatin (**c**) or 800 μM capecitabine (**d**) for 24 h. The cell apoptosis was detected by flow cytometry. The IC50 values from the CCK-8 assay were calculated to assess the sensitivity to cisplatin (**e**) and capecitabine (**f**) in control and CASC2 overexpressing KYSE30 and KYSE150 cells. The IC50 values from the CCK-8 assay were calculated to assess the sensitivity to cisplatin (**g**) and capecitabine (**h**) in control and CASC2 knockdwon KYSE30 and KYSE150 cells. Error bars indicate SD. *p < 0.05

We then determined whether CASC2 played a role in drug resistance in ESCC cells. Apoptosis assays by flow cytometric analysis were used to test the effect of CASC2 on the chemotherapeutic drug sensitivity of ESCC cells. We found that CASC2 overexpression significantly enhanced apoptosis of KYSE30 and KYSE150 cells treated with cisplatin or capecitabine for 24 h (Fig. 3a, b, Additional file 2: Figure S2A, B). Conversely, cell apoptosis rate of KYSE30 and KYSE150 cells treated with cisplatin or capecitabine were notably inhibited by CASC2 knockdown compared to control groups (Fig. 3c, d, Additional file 2: Figure S2C and D). Moreover, we detected the IC50 (half maximal inhibitory concentration) changes to cisplatin or capecitabine after CASC2 alteration in ESCC cells. ESCC cells were treated with continuous concentrations of cisplatin or capecitabine for 48 h, and then the cell viability was tested by CCK-8 assays. The IC50 of cisplatin or capecitabine in CASC2-overexpressed KYSE30 and KYSE150 cells were much lower compared to control groups (Fig. 3e, f, Additional file 2: Figure S2E and F), while knockdown of CASC2 significantly increased the IC50 of cisplatin or capecitabine (Fig. 3g, h, Additional file 2: Figure S2G and H). Collectively, these data suggested that CASC2 could regulate the sensitivity of ESCC cells in response to chemotherapeutics.

### CASC2 upregulates SOCS1 expression

**Fig. 4:**
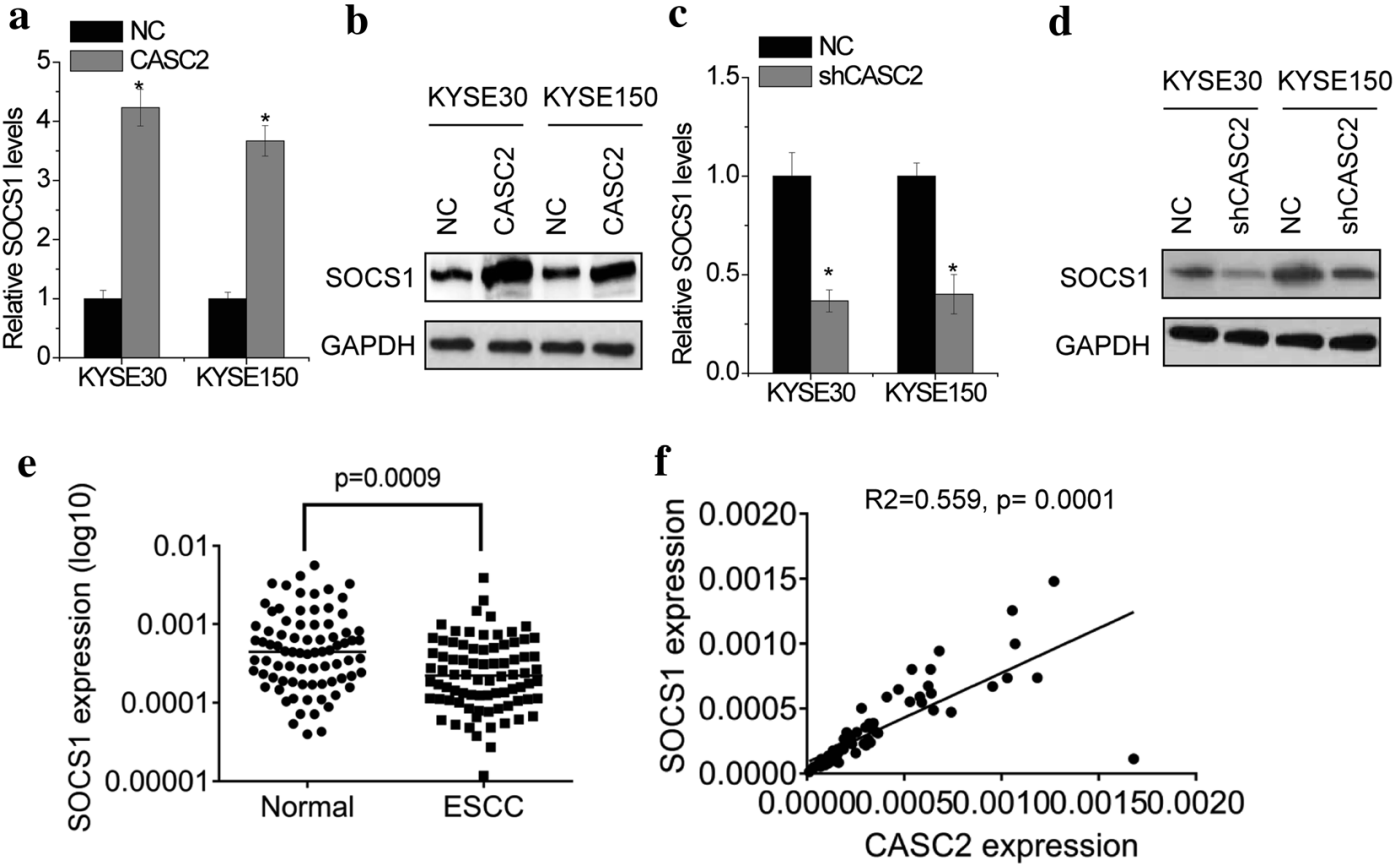
CASC2 upregulates SOCS1 expression. The mRNA (**a**) and protein (**b**) level of SOCS1 in control and CASC2 overexpressing KYSE30 and KYSE150 cells was detected by real-time PCR and western blot, respectively. The mRNA (**c**) and protein (**d**) levels of SOCS1 in control and CASC2 knockdown KYSE30 and KYSE150 cells was detected by real-time PCR and western blot, respectively. **e** The mRNA expression levels of SOCS1 in 78 pairs of ESCC tissues and adjacent normal tissues were detected by using real-time PCR. **f** The Pearson correlation analyses were performed to analyze the correlation between CASC2 and SOCS1 expression in ESCC tissues. Error bars indicate SD. *p < 0.05

To investigate the potential genes involved in CASC2 function, we used an RNA-sequencing analysis in KYSE30 cells with or without CASC2 overexpression. Among these genes, we found that the tumor suppressor Suppressors of Cytokine Signaling 1 (SOCS1) was one of the most elevated genes regulated by CASC2 overexpression (Additional file 3: Figure S3). For validation of these results, qRT-PCR and western blot was conducted in ESCC cells with CASC2 alteration. The results showed that CASC2 overexpression significantly increased both mRNA and protein levels of SOCS1 (Fig. 4a, b). In contrast, depletion of CASC2 could downregulate SOCS1 expression in KYSE30 and KYSE 150 cells (Fig. 4b, c). The SOCS1 mRNA levels in the same set of 78 pairs of ESCC tissues and adjacent normal tissues were assessed using real-time PCR. The results showed that SOCS1 mRNA levels were markedly downregualted in ESCC tissues compared to normal tissues (Fig. 4e). Moreover, a correlation analysis showed that CASC2 expression positively correlated with SOCS1 expression in ESCC tissues (Fig. 4e, R^2^ = 0.559, p = 0.0001). Therefore, we selected SOCS1 which was involved in cancer cell proliferation, migration, invasion and drug resistance [15, 16], as a potential CASC2 target.

### CASC2 acts as a ceRNA for miR-155 to regulate SOCS1 expression

**Fig. 5:**
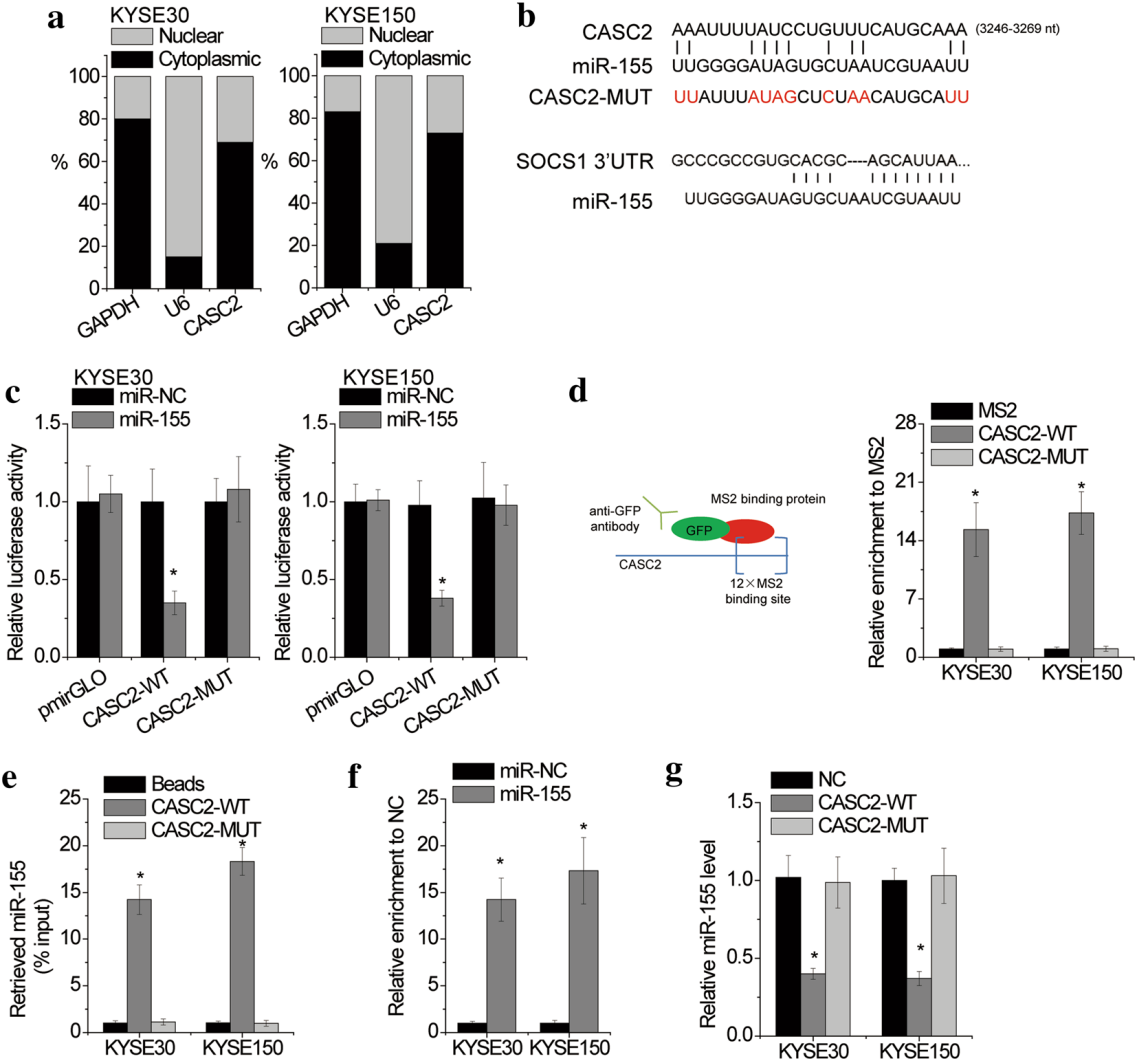
CASC2 interacts with miR-155. **a** The subcellular location of CASC2 in KYSE30 and KYSE150 cells. **b** The prediction for miR-155 binding sites on CASC2 transcript and SOCS1 3′UTR. **c** Luciferase activity in KYSE30 and KYSE150 cells transfected with miR-155 mimics and luciferase reporters containing nothing, wild-type CASC2 (CASC2-WT) or mutant CASC2 (CASC2-MUT). Data are presented as the relative ratio of firefly luciferase activity to renilla luciferase activity. The relative values was normalized to miR-NC group. **d** MS2-RIP followed by real-time PCR to detect miR-155 endogenously associated with CASC2. **e** KYSE30 and KYSE150 cell lysates were incubated with biotin-labeled wild-type or mutant CASC2; after pull-down, miR-155 was assessed by real-time PCR. **f** Anti-AGO2 RIP was performed in KYSE30 and KYSE150 cells transfected with miR-155 mimics, followed by real-time PCR to detect CASC2 associated with AGO2. **g** The miR-155 expression in KYSE30 and KYSE150 cells with overexpression of wild-type CASC2 or mutant CASC2. Error bars indicate SD. *p < 0.05

To elucidate the underlying molecular mechanism of CASC2, we first detected the subcellular localization of CASC2 using real-time PCR and found that CASC2 was primarily located in the cytoplasm of KYSE30 and KYSE150 cells (Fig. 5a), suggesting that CASC2 may exert its function as a competing endogenous RNA (ceRNA). A bioinformatics analysis predicted that CASC2 harboured binding sequences of the miR-155 (Fig. 5b) which was reported to target SOCS1 mRNA [17, 18]. The sequences of CASC2 with the miR-155 binding site and a mutation (CASC2-MUT) were inserted into the luciferase reporter vector pmirGLO for the luciferase assay. As shown in Fig. 5c, compared with the control vector, the luciferase activities were significantly decreased by miR-155 overexpression in the wild-type (CASC2-WT) reporter vector, but not in empty vector or mutant reporter vector. To further confirm the interaction between CASC2 and miR-155, we performed an RNA immunoprecipitation (RIP) assay to pull down endogenous microRNAs associated with CASC2. MS2-binding protein (MS2 bp) specifically binds RNA containing MS2-binding sequences (MS2bs). The pcDNA3.1 plasmid containing CASC2 or CASC2-MUT combined with MS2bs elements was constructed and then cotransfected into cells with a plasmid expressing MS2 bp-GFP. The RIP assay was then performed using anti-GFP antibody. Of note, the CASC2 RIP in both KYSE30 and KYSE150 cells was significantly enriched for miR-155 compared to the empty vector (MS2) and CASC2 with mutations in miR-155 targeting sites (CASC2-MUT) (Fig. 5d). The results of RNA pull-down assays further demonstrated the specific association between miR-155 and CASC2 (Fig. 5e). Additionally, we conducted anti-AGO2 RIP in KYSE30 and KYSE150 cells transfected with miR-155 mimics. The results showed that endogenous CASC2 pull-down by AGO2 was specifically enriched in miR-155-transfected cells (Fig. 5f). Overexpression of wild-type CASC2, but not the mutant, led to a decrease in miR-155 expression (Fig. 5g). Together, all these data suggested sequence-specific binding of miR-155 to CASC2.

**Fig. 6:**
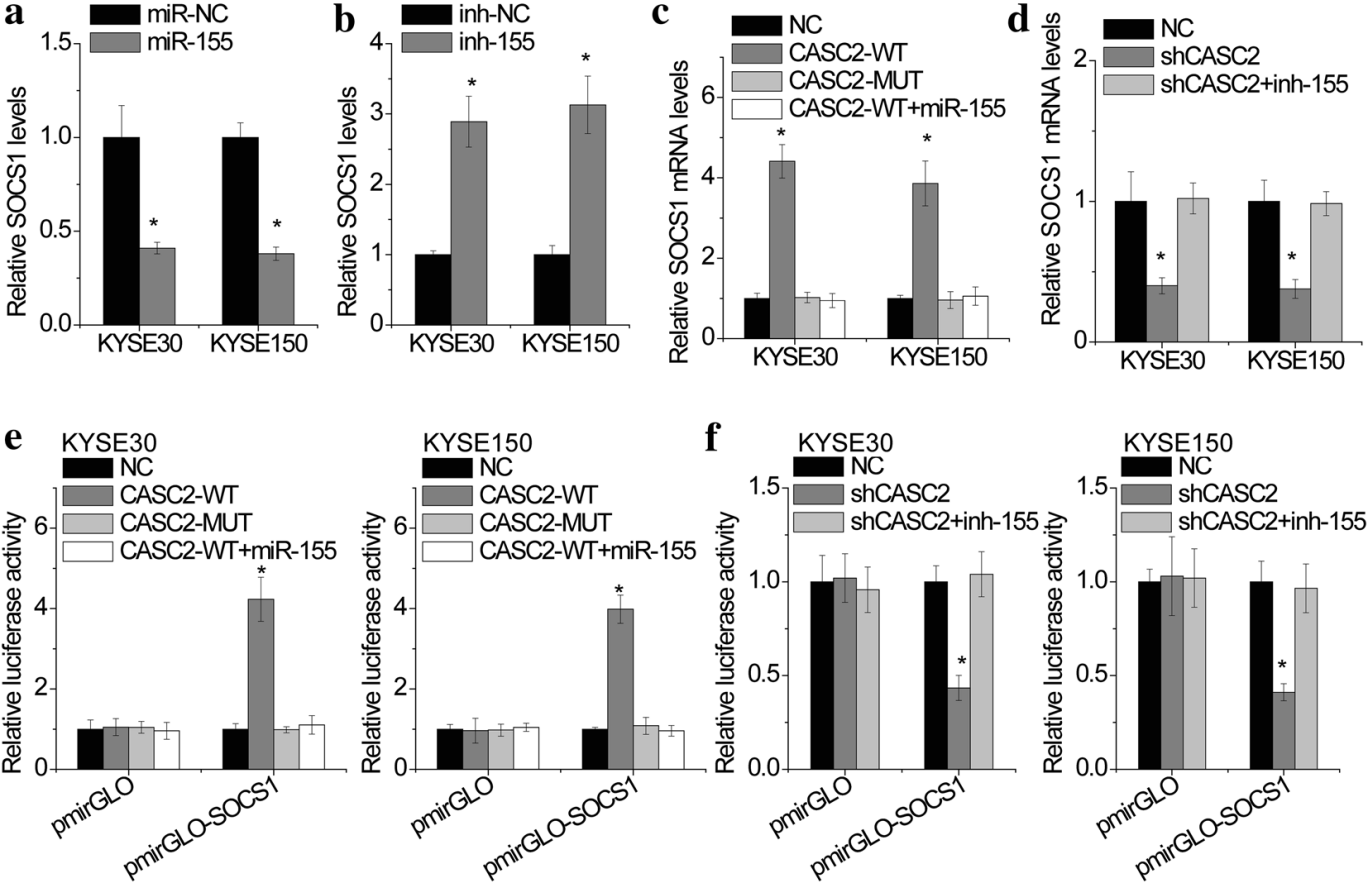
CASC2 upregulates SOCS1 by competitively binding miR-155. **a** KYSE30 and KYSE150 cells were transfected with miR-155 mimics and the expression of SOCS1 mRNA was detected by real-time PCR. **b** KYSE30 and KYSE150 cells were transfected with miR-155 inhibitor and the expression of SOCS1 mRNA was detected by real-time PCR. **c** miR-155 mimics abolished the upregulation of SOCS1 expression mediated by CASC2 overexpression. **d** miR-155 inhibitor abolished the downregulation of SOCS1 expression mediated by CASC2 knockdown. **e** Luciferase activity in CASC2 overexprsessing KYSE30 and KYSE150 cells co-transfected with miR-155 mimics and luciferase reporters containing SOCS1 3′UTR. **f** Luciferase activity in CASC2 silencing KYSE30 and KYSE150 cells co-transfected with miR-155 inhibitor and luciferase reporters containing SOCS1 3′UTR. Error bars indicate SD. *p < 0.05

We then determined whether CASC2 regulated SOCS1 expression in a miR-155-dependent manner. miR-155 overexpression significantly decreased SOCS1 expression, whereas depletion of miR-155 upregulated SOCS1 expression (Fig. 6a, b), indicating that SOCS1 was a target of miR-155. miR-155 mimics were transfected into CASC2-overexpressed KYSE30 and KYSE150 cells. Overexpression of wild-type CASC2, but not the mutant, upregulated SOCS1 expression, while miR-155 transfection abrogated this increase (Fig. 6c). Conversely, CASC2-silenced KYSE30 and KYSE150 cells were transfected with miR-155 inhibitor. The SOCS1 downregulation mediated by CASC2 knockdown was abolished by inhibition of miR-155 (Fig. 6d).

To ascertain whether this observed effect depends on regulation of the SOCS1 3′UTR, we constructed luciferase reporters containing SOCS1 3′UTR (pmirGLO-SOCS1). pmirGLO or pmirGLO-SOCS1 luciferase plasmid was transfected into KYSE30 and KYSE150 cells with CASC2 overexpression or knockdown. We found that overexpression of wild-type CASC2, but not the mutant, significantly increased the luciferase activity of pmirGLO-SOCS1, while transfection of miR-155 mimics abolished this effect (Fig. 6e). In contrast, CASC2 knockdown decreased the luciferase activity of pmirGLO-SOCS1, which was attenuated by inhibition of miR-155 (Fig. 6f). Collectively, these data demonstrated that CASC2 upregulated SOCS1 expression by competitively binding miR-155.

### CASC2 interacts with and stabilizes SOCS1

**Fig. 7:**
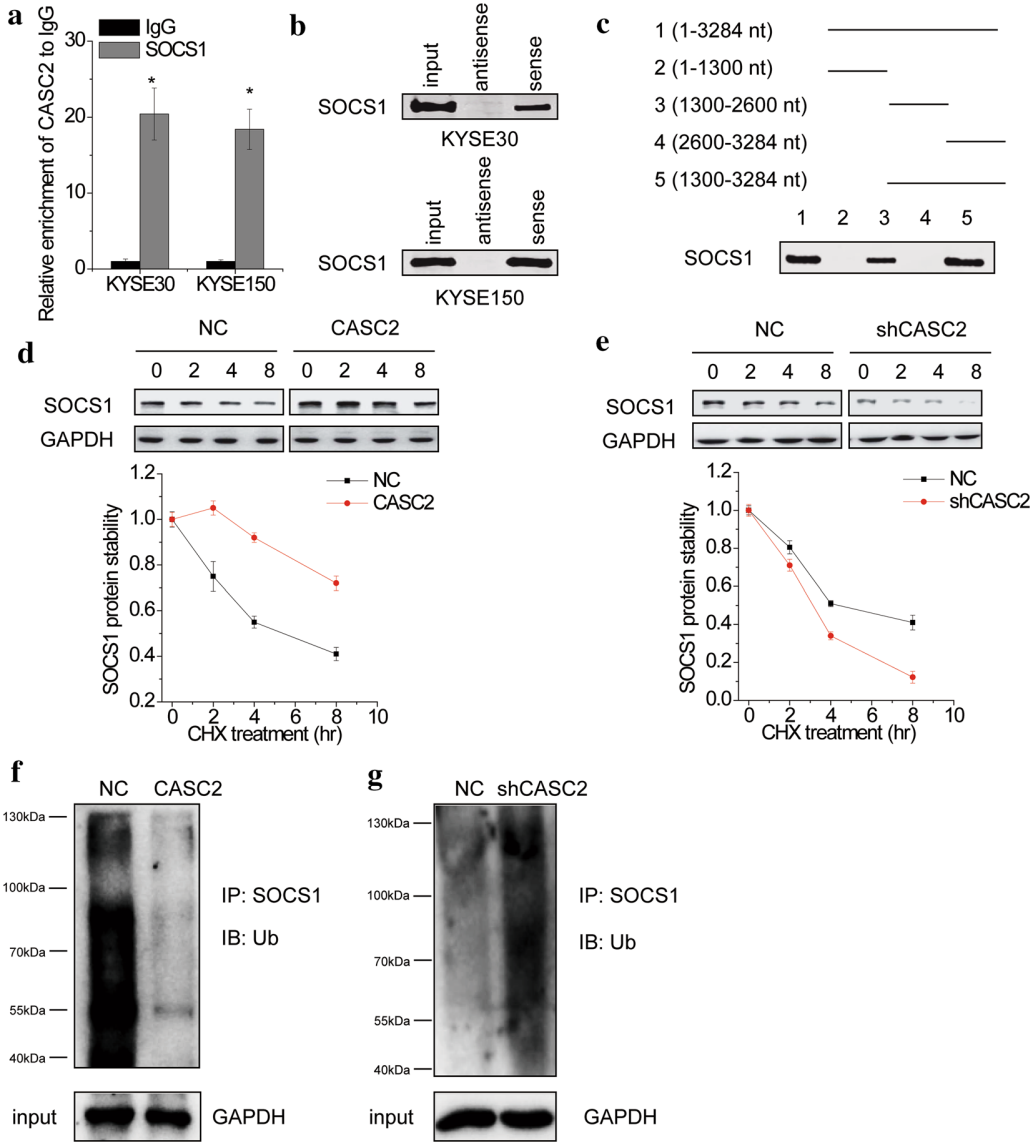
CASC2 enhances stability of SOCS1 protein. **a** RIP followed by real-time PCR assay was used to analyze the interaction between CASC2 and SOCS1 protein. Relative quantification of CASC2 in RNA–protein complexes immunoprecipitated with IgG or SOCS1 antibodies from cell extracts. IgG was taken as a negative control. **b** Western blot of SOCS1 expression in protein complexes pulled down by full-length CASC2 or its antisense control fragment. **c** Deletion mapping of SOCS1-binding domain in CASC2. Western blot of SOCS1 in protein samples pulled down by different CASC2 fragments. **d** Control and CASC2 overexpressing KYSE30 cells were treated with CHX (100 μg/ml) for the indicated time points. The cell lysates were examined by immunoblotting. A plot of normalized amount of SOCS1 protein is shown. **e** Control and CASC2 knockdown KYSE150 cells were treated with CHX (100 μg/ml) for the indicated time points. The cell lysates were examined by immunoblotting. A plot of normalized amount of SOCS1 protein is shown. **f** Control and CASC2 overexpressing KYSE30 cell lysates were immunoprecipitated with anti-SOCS1 antibody, and the immunocomplexes were immunoblotted with antibodies against Ub. **g** Control and CASC2 knockdown KYSE150 cell lysates were immunoprecipitated with anti-SOCS1 antibody, and the immunocomplexes were immunoblotted with antibodies against Ub. Error bars indicate SD. *p < 0.05

We then detected whether CASC2 could interact with SOCS1 by performing RIP assay. Interestingly, as shown in Fig. 7a, SOCS1 could be significantly pulled down by the anti-SOCS1 antibody compared to the negative control IgG in both KYSE30 and KYSE150 cells. To further validate the interaction between CASC2 and SOCS1, we subjected the CASC2-pull-down protein samples to immunoblotting with SOCS1 antibody. A strong signal was observed in proteins pulled down with CASC2 RNA but not in samples bound with negative control antisense CASC2 (Fig. 7b). Furthermore, using a series of deletion-mapping analyses, we identified that a 1300 nt region in the middle of the CASC2 transcript (1300–2600 nt) is required for CASC2-SOCS1 association (Fig. 7c).

Next, we explored the molecular consequences of the association between lncRNA CASC2 and SOCS1. Previous studies reported that lncRNA could regulate the stability of its interacting proteins [19–21]. To test whether CASC2 affected the stability of SOCS1 protein, we examined the effect of both CASC2 depletion and overexpression on the half-life of of endogenous SOCS1 protein in the presence of the inhibitor of protein translation, cycloheximide (CHX). The results indicated that CASC2 overexpression significantly prolonged the half-life of SOCS1 protein in KYSE30 cells (Fig. 7d). In contrast, knockdown of CASC2 dramatically promoted the degradation of SOCS1 in KYSE150 cells (Fig. 7e). In agreement with this observation, overexpression of CASC2 significantly suppressed the level of SOCS1 ubiquitination in KYSE30 cells (Fig. 7f), while CASC2 knockdown increased the level of SOCS1 ubiquitination in KYSE150 cells (Fig. 7g). These data confirmed that CASC2 inhibited the proteasome-mediated degradation of SOCS1.

**Fig. 8:**
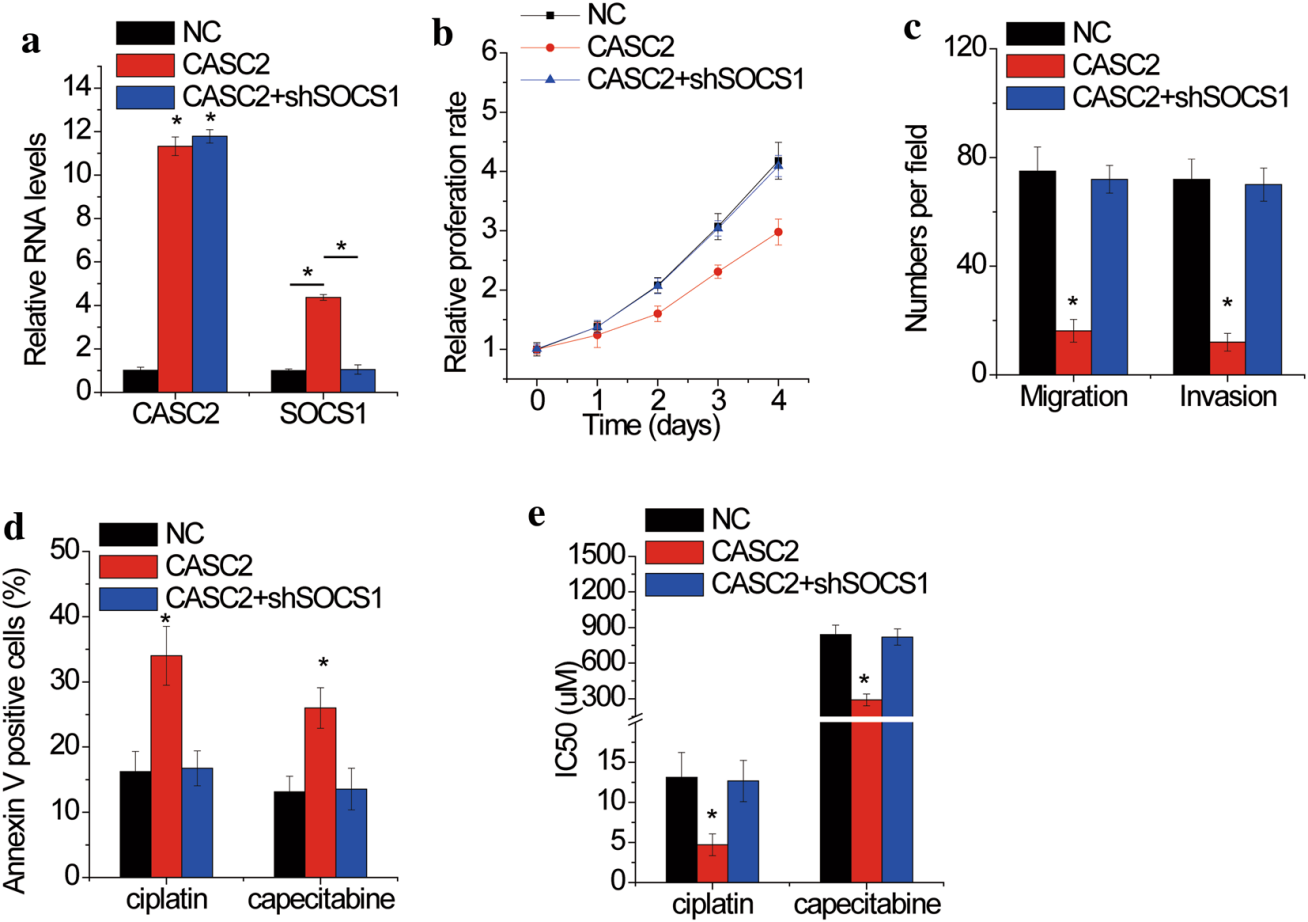
CASC2 exerts tumor suppressive effects through SOCS1. **a** SOCS1 expression was silenced in CASC2 overexpressing KYSE30 cells. **b** The proliferative abilities of KYSE30 cells after co-transfection with CASC2 and SOCS1 shRNA were determined by CCK-8 assay. **c** The migration and invasion abilities of KYSE30 cells after co-transfection with CASC2 and SOCS1 shRNA were determined by transwell assay. **d** CASC2 overexpressing KYSE30 cells transfected with SOCS1 shRNA were treated with 10 μM cisplatin or 800 μM capecitabine for 24 h. The cell apoptosis was detected by flow cytometry. **e** The IC50 values from the CCK-8 assay were calculated to assess the sensitivity to cisplatin and capecitabine in control and CASC2 overexpressing KYSE30 cells transfected with SOCS1 shRNA. Error bars indicate SD. *p < 0.05

We finally determined whether SOCS1 was involved in the functions of CASC2 in regulating cell proliferation, migration, invasion and drug sensitivity. CASC2-overexpressed KYSE30 cells were transfected with SOCS1 shRNA (Fig. 8a). We found that the suppression of proliferation, migration and invasion induced by CASC2 overexpression was abolished by SOCS1 depletion (Fig. 8b, c). Moreover, silence of SOCS1 attenuated the CASC2-mediated sensitivity of KYSE30 cell to cisplatin or capecitabine (Fig. 8d, f). These results indicated that CASC2 exerted suppressive effects through SOCS1.

## Discussion

Some lncRNAs have emerged as critical regulators in ESCC tumorigenesis and development. However, only a handful of tumor suppressive lncRNAs has been well demonstrated. Here, we found that CASC2 expression was significantly reduced in ESCC tissues. Downregulation of CASC2 expression was associated with tumor differentiation, lymph node metastasis, and TNM stage, and also predicted a shorter overall time in patients with ESCC. Performing knockdown and overexpression assays, we demonstrated that the CASC2 inhibited ESCC cell proliferation, migration and invasion, as well as promoted the sensitivity against cisplatin and capecitabine. Our findings suggested that CASC2 functions as a tumor suppressor in ESCC, and its downregulation contributes to ESCC progression.

LncRNAs exert their functions through diverse mechanisms, including transcriptional regulation, scaffolding of nuclear or cytoplasmic complexes, post-transcriptional regulation and post-translational modification [22, 23]. Emerging evidence has revealed that some lncRNAs can serve as microRNA “sponges” by sharing common microRNA recognition elements (MREs) [24, 25]. The interactions between lncRNAs and microRNAs could inhibit the activity of microRNAs, and suppress the inhibition of microRNAs in target mRNAs. Previous studies have shown that CASC2 could function as microRNA sponge. For instance, in malignant melanoma cells, CASC2 associates with miR-18a and then upregulates RUNX1 expression, subsequently suppressing cell proliferation, migration and invasion [26]. In cisplatin-resistant cervical cancer cells, CASC2 upregulates PTEN expression by directly inhibiting miR-21 and inactivation of AKT signaling [27]. Oncogenic miR-21 can bind to CASC2 in a sequence-specific manner and abrogate CASC2-mediated inhibition of proliferation, migration, and invasion in glioma cells [28]. Moreover, the expression of CASC2 is significantly decreased in breast cancer tissues. CASC2 overexpression inhibites the viability, migration and invasion, and elevates apoptosis of breast cancer cells through acting as a ceRNA for miR-96 and upregulating synoviolin (SYVN1) expression [11]. miR-155 has been found to be overexpressed in ESCC tissues and exerts as an oncogene to increase the proliferation and colonies formation of ESCC cells through targeting p53-induced nuclear protein 1 (TP53INP1) [29]. Here, for the first time, our results of RIP, RNA pull-down and luciferase activity demonstrated a direct interaction between CASC2 and miR-155. Moreover, we revealed that CASC2 functioned as a ceRNA of tumor suppressor SOCS1 against miR-155. Our findings highlighted the role of CASC2 as a ceRNA in regulating ESCC progression.

SOCS1 was recognized as a negative feedback regulator of cytokine signaling, such as the Janus kinases–signal transducers and activators of transcription (JAK-STAT) signaling pathway, which is important in cellular activation, proliferation, metastasis, differentiation, and drug resistance [30]. Loss of SOCS1 has been observed in human cancers, including hepatocellular carcinoma, breast cancer, acute myeloid leukemia, and ESCC [31–34]. Several studies found that the promoters of *SOCS1* gene were often hypermethylation in some human cancers [34–36]. miRNAs also play a role in SOCS1 silence. It was found that miR-221 could regulate SOCS1 expression through targeting its 3′UTR, thus promoting proliferation and migration of prostate cancer cells in vitro and facilitating tumorigenesis in vivo [37]. Another group found that miR-155 could regulate SOCS1 expression by the same mechanism [38]. Here, we showed that lncRNA CASC2 was involved in the dysregulation of SOCS1 in ESCC cells. CASC2 upregulated SOCS1 by competitively binding miR-155. Besides, we also identified a direct interaction between CASC2 and SOCS1 protein. CASC2 associated with SOCS1 protein and suppressed its ubiquitination level, which led to an increase of SOCS1 protein stability. A previous study demonstrated that SOCS1 interacted with members of the Pim family of serine/threonine kinases in thymocytes. Coexpression of the Pim kinases with SOCS1 results in stabilization of the SOCS1 protein [39]. Whether Pim family plays a role in CASC2-mediated SOCS1 stabilization needs further exploration. Here, we speculated that the ceRNA mechanism was more prominent in CASC2-mediated SOCS1 upregulation. The change of SOCS1 protein induced by CASC2 was only slightly higher than SOCS1-mediated SOCS1 mRNA change (Fig. 4a–d), indicating that CASC2-mediated miR-155 suppression was more important for the SOCS1 elevation. Moreover, miR-155 could target other oncogenes in ESCC, which suggested that the functional role of CASC2 as a miR-155 sponge is more prominent in regulating ESCC aggressiveness.

## Conclusion

In conclusion, our research demonstrated that CASC2 acted as an important tumor suppressor in ESCC progression. CASC2 upregulated SOCS1 expression through two different mechanisms: CASC2 upregulated SOCS1 by competitively binding miR-155 and stabilizing SOCS1 protein. The protective effects of CASC2 on ESCC progression imply that CASC2 has potential use in ESCC treatment and needs further investigation.

## Supplementary information

**Additional file 1: Figure S1.** CASC2 inhibites ESCC cell proliferation. A. The represent images of Fig. 2d. B. The represent images of Fig. 2g.

**Additional file 2: Figure S2.** CASC2 promotes the drug sensitivity of ESCC cells. A and B. Control and CASC2 overexpressing KYSE30 and KYSE150 cells were treated with 10 μM cisplatin (A) or 800 μM capecitabine (B) for 24 h. The cell apoptosis was detected by flow cytometry. C and D. Control and CASC2 knockdown KYSE30 and KYSE150 cells were treated with 10 μM cisplatin (C) or 800 μM capecitabine (D) for 24 h. The cell apoptosis was detected by flow cytometry. E and F. CCK-8 assay was used to assess the sensitivity to cisplatin (E) and capecitabine (F) in control and CASC2 overexpressing KYSE30 and KYSE150 cells. G and H. CCK-8 assay was used to assess the sensitivity to cisplatin (G) and capecitabine (H) in control and CASC2 knockdwon KYSE30 and KYSE150 cells.

**Additional file 3.** The top differentially expressed genes regulated by CASC2 overexpression.

## Data Availability

The datasets used during this research are available.

## Abbreviations

ESCC: esophageal squamous cell carcinoma
lncRNAs: long noncoding RNAs
CASC2: Cancer Susceptibility Candidate 2
ceRNA: competing endogenous RNA
PART1: Prostate Androgen-Regulated Transcript 1
YBX1: Y-Box Binding Protein 1
RIP: RNA immunoprecipitation
MREs: microRNA recognition elements
SYVN1: synoviolin
TP53INP1: p53-induced nuclear protein 1
JAK-STAT: Janus kinases-signal transducers and activators of transcription
