# Supplementary figures and images for: Long noncoding RNA CASC2 suppresses esophageal squamous cell carcinoma progression by increasing SOCS1 expression
Source: Cell Biosci. 2019 Nov 9;9:90. doi: 10.1186/s13578-019-0353-4 (PMC6842511; doi:10.1186/s13578-019-0353-4)

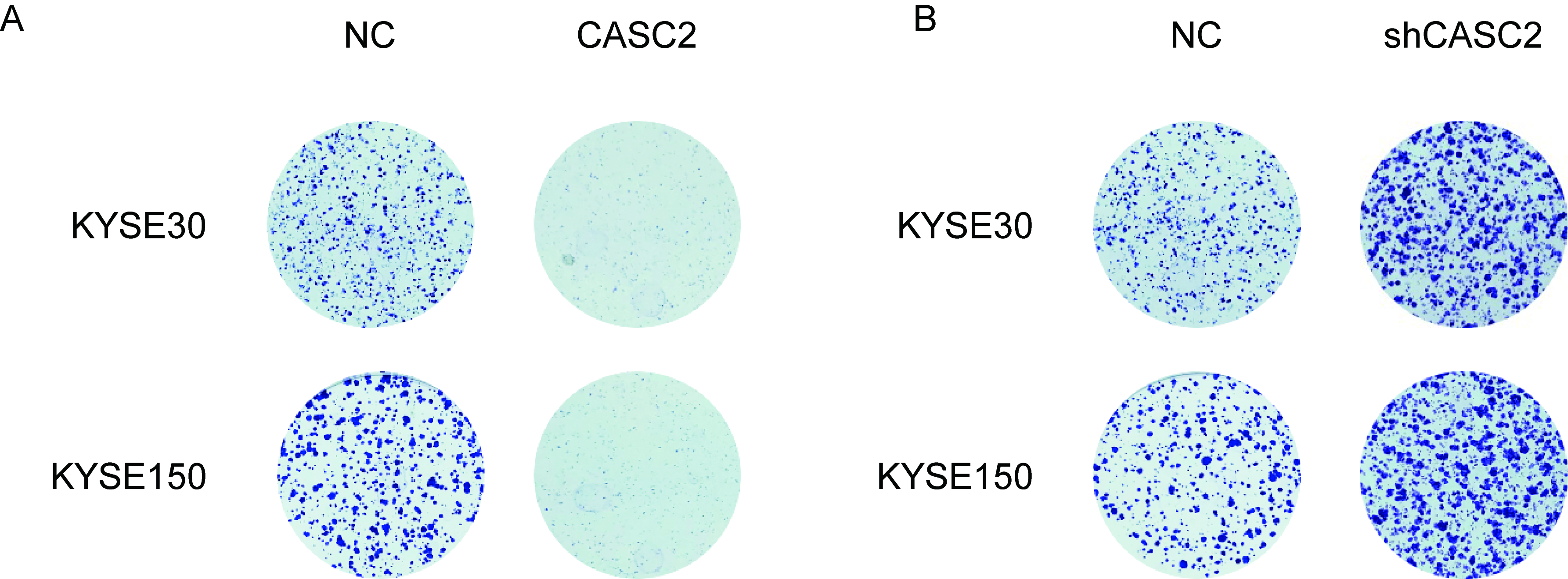

Supplement: Supplementary file 1 — Additional file 1: Figure S1. CASC2 inhibites ESCC cell proliferation. A. The represent images of Fig. 2d. B. The represent images of Fig. 2g. [file 13578_2019_353_MOESM1_ESM.tif]

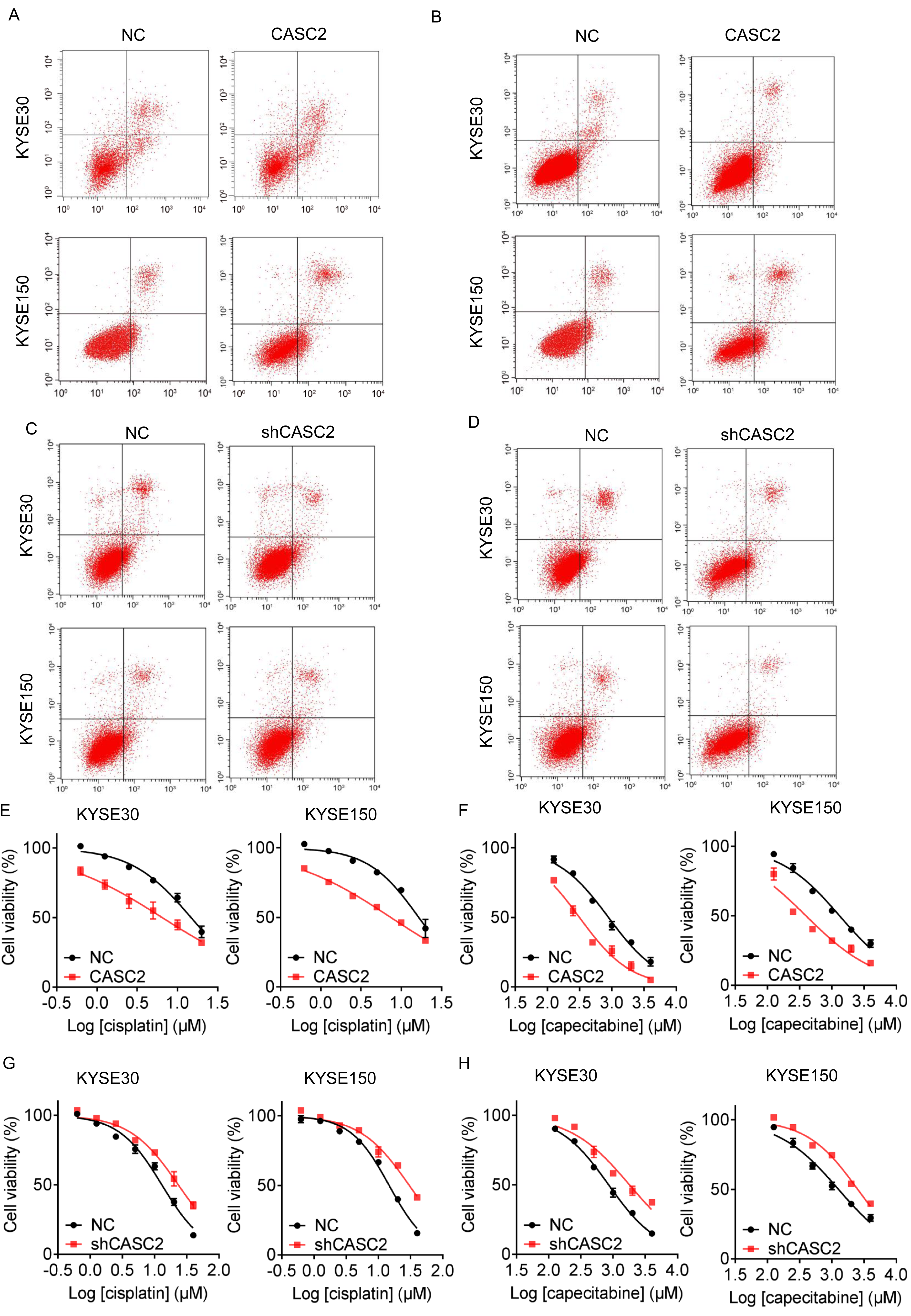

Supplement: Supplementary file 2 — Additional file 2: Figure S2. CASC2 promotes the drug sensitivity of ESCC cells. A and B. Control and CASC2 overexpressing KYSE30 and KYSE150 cells were treated with 10 μM cisplatin (A) or 800 μM capecitabine (B) for 24 h. The cell apoptosis was detected by flow cytometry. C and D. Control and CASC2 knockdown KYSE30 and KYSE150 cells were treated with 10 μM cisplatin (C) or 800 μM capecitabine (D) for 24 h. The cell apoptosis was detected by flow cytometry. E and F. CCK-8 assay was used to assess the sensitivity to cisplatin (E) and capecitabine (F) in control and CASC2 overexpressing KYSE30 and KYSE150 cells. G and H. CCK-8 assay was used to assess the sensitivity to cisplatin (G) and capecitabine (H) in control and CASC2 knockdwon KYSE30 and KYSE150 cells. [file 13578_2019_353_MOESM2_ESM.tif]

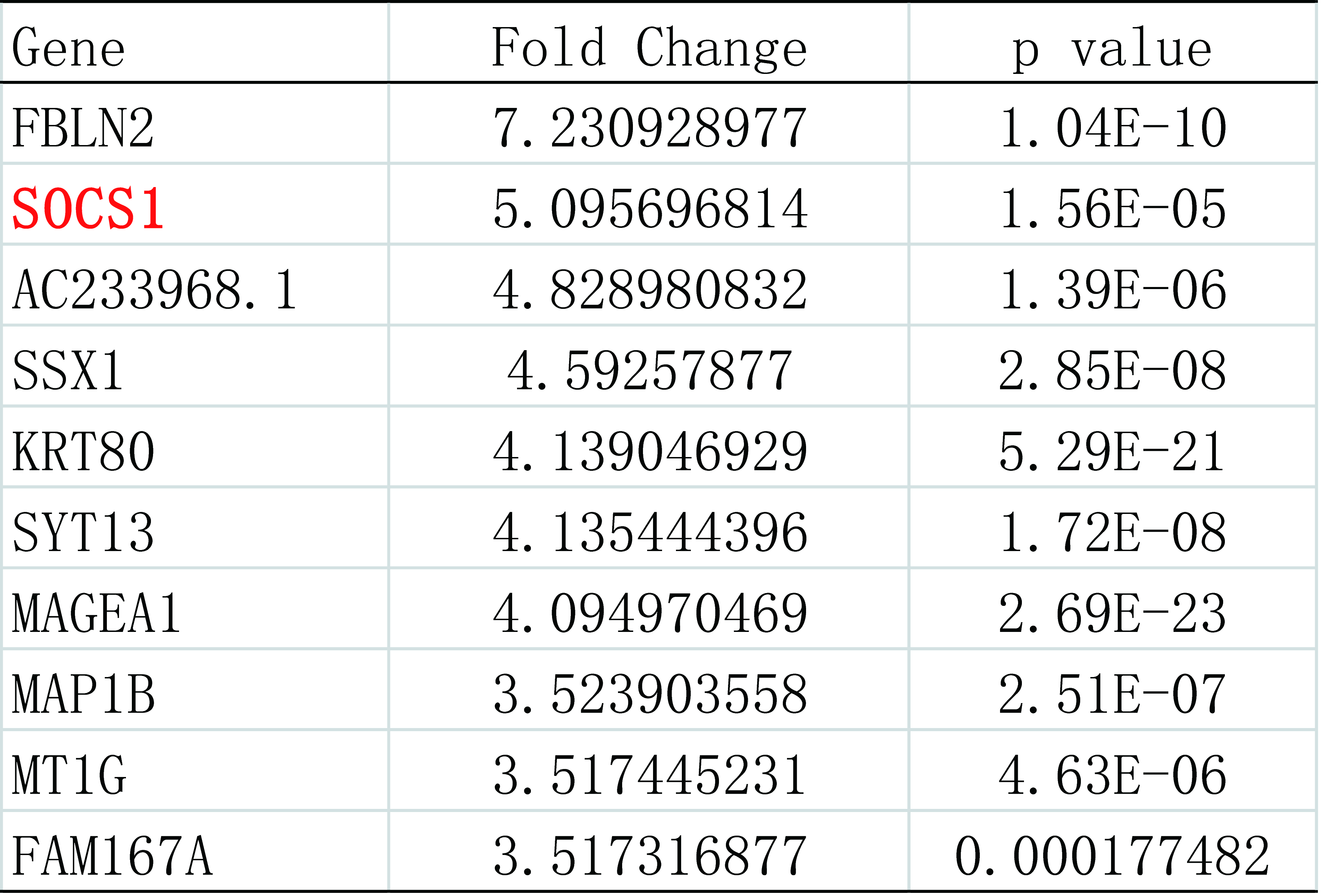

Supplement: Supplementary file 3 — Additional file 3. The top differentially expressed genes regulated by CASC2 overexpression. [file 13578_2019_353_MOESM3_ESM.tif]
